# Fatal Cardiac Tamponade: The Lethal Progression of Acute-on-Chronic Pericardial Effusion

**DOI:** 10.7759/cureus.62950

**Published:** 2024-06-23

**Authors:** Bernard Brown, Rita Offor, Bisrat Nigussie, Suzette Graham-Hill

**Affiliations:** 1 Internal Medicine, Downstate Health Sciences University, Brooklyn, USA; 2 Cardiology, Downstate Health Sciences University, Brooklyn, USA; 3 Cardiology, Kings County Hospital Center, Brooklyn, USA

**Keywords:** pericardial effusion, cardiac tamponade, malignant pericardial effusion, end-stage renal disease (esrd), recurrent pericardial effusion, pericardial effusion with cardiac tamponade, impending cardiac tamponade

## Abstract

Cardiac tamponade is a life-threatening occurrence with an incidence rate of about two out of 1,000 people. It is caused by the rapid accumulation of fluid in the pericardial sac. This can lead to the physical examination findings of tachycardia, hypotension, and elevated jugular venous pressure. Patients with chronic pericardial effusion are at increased risk for cardiac tamponade. We present a case of a patient with chronic, recurrent, malignant pericardial effusion that rapidly evolved to cardiac tamponade several hours from hospital presentation. We attempt to highlight the importance of close monitoring of patients who have recurrent chronic pericardial effusion in hopes of decreasing the number of patients who develop cardiac tamponade physiology.

## Introduction

Pericardial effusion refers to the buildup of fluid in the pericardial sac surrounding the heart, which can stem from diverse factors, such as end-stage renal disease, cancer, systemic lupus erythematosus, myocardial infarction, aortic dissection, infections, traumatic injury, and medications, among others [1]. Cardiac tamponade poses a life-threatening risk and arises from the rapid or recurrent progression of pericardial effusion, where the fluid accumulation compresses the heart, impairing its pumping function [2,3]. Immediate recognition and intervention are imperative in managing cardiac tamponade. Indications of this condition encompass tachycardia, hypotension, elevated jugular venous pressure, muffled heart sounds, and pulsus paradoxus [3]. Treatment modalities for cardiac tamponade involve addressing or managing the underlying cause of effusion, along with interventions such as pericardiocentesis or surgical pericardiotomy [3].

## Case presentation

**Figure 1:**
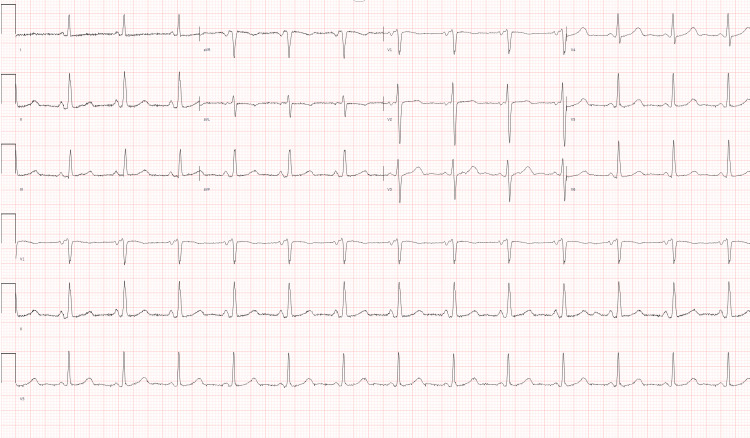
Electrocardiogram depicting normal sinus rhythm at 80 beats per minute.

**Figure 2:**
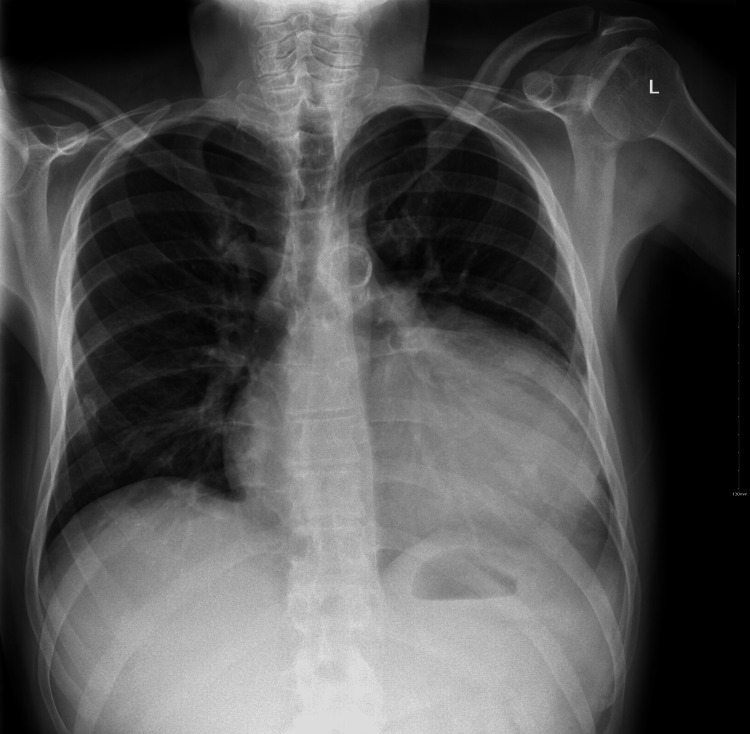
Chest X-ray depicting enlargement of the cardiac silhouette.

**Figure 3:**
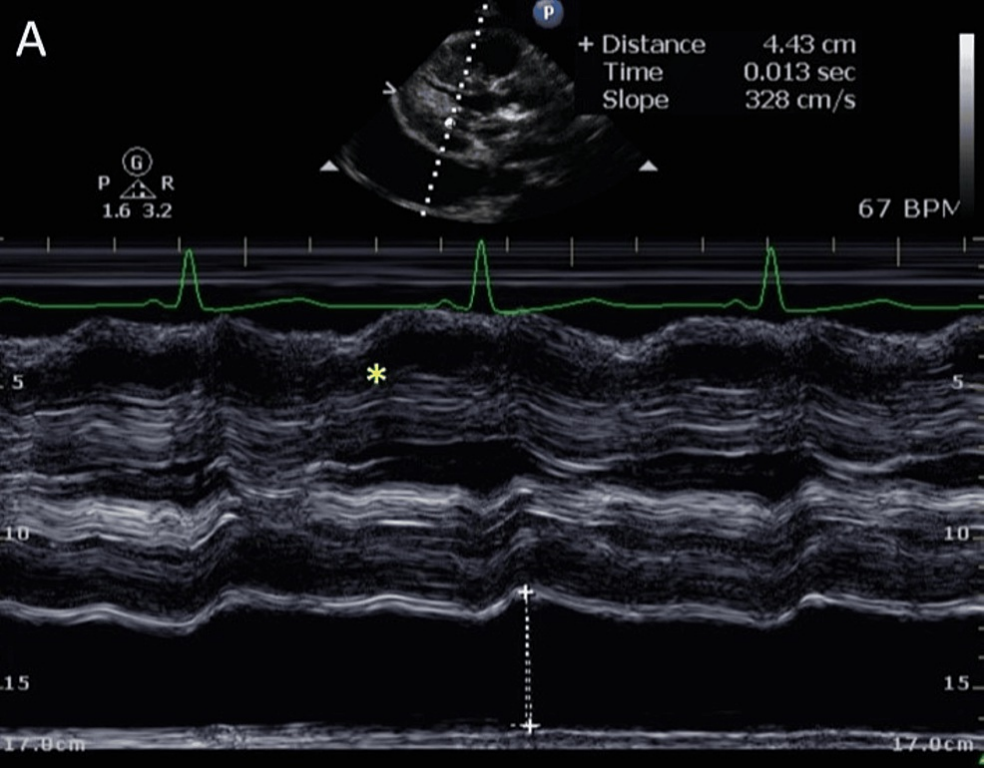
Transthoracic echocardiogram M-mode depicting right ventricle collapse in diastole (yellow star).

**Figure 4:**
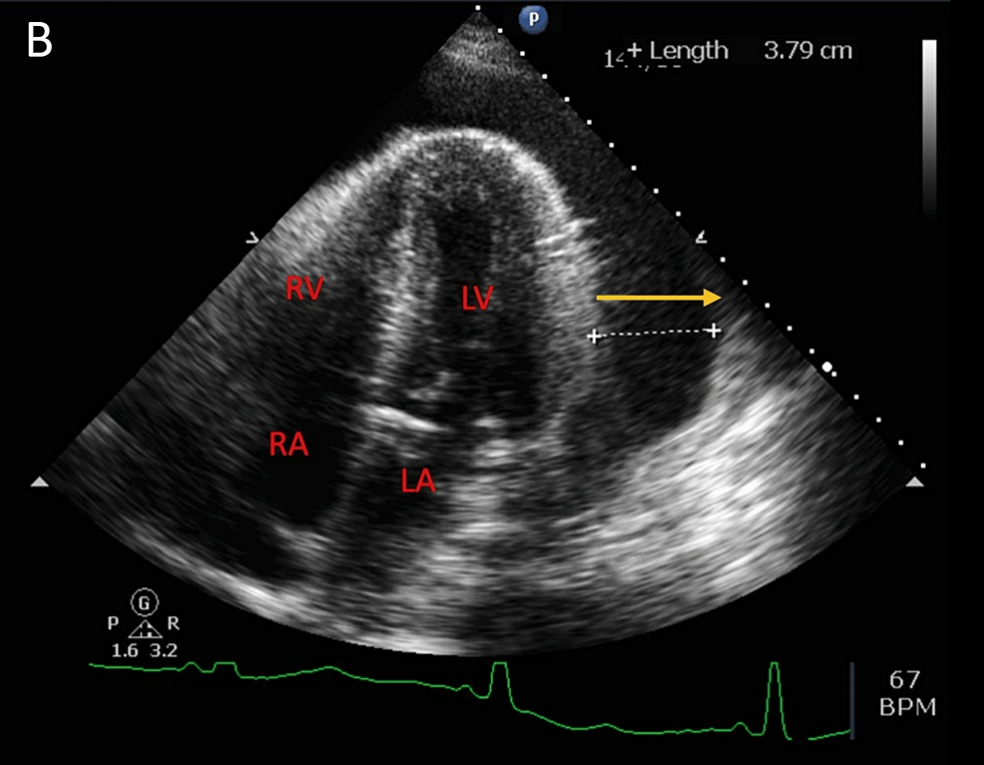
Transthoracic echocardiogram four-chamber view depicting large volume pericardial effusion (yellow arrow). LV: left ventricle; LA: left atrium; RV: right ventricle; RA: right atrium.

**Figure 5:**
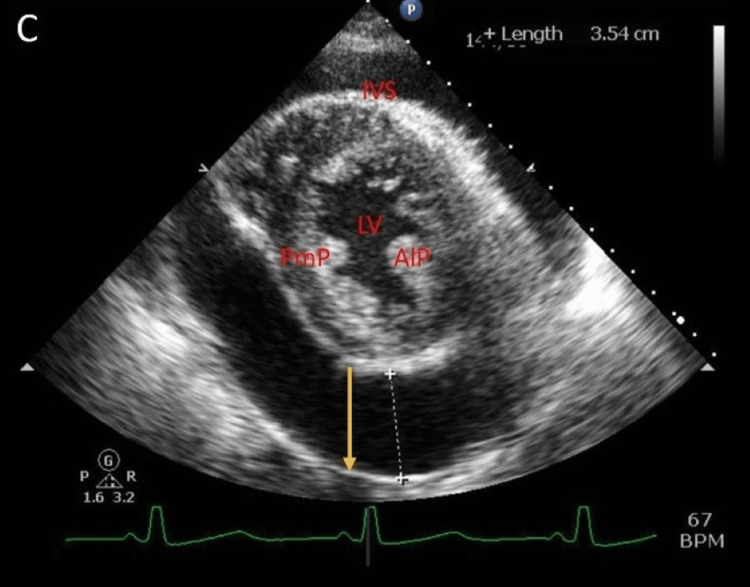
Transthoracic echocardiogram parasternal short-axis view with large pericardial effusion (yellow arrow). PmP: posteromedial papillary muscle; AIP: anterolateral papillary muscle; IVS: interventricular septum; LV: left ventricle.

A 51-year-old male with a medical history of cerebral palsy, hypertension, subclinical hypothyroidism, thyroid disease, and end-stage renal disease on hemodialysis presented to the emergency department (ED). His chief complaints were generalized weakness, fatigue, and right shoulder pain. The symptoms had been present for one day, exacerbated after recent hemodialysis. Physical examination revealed a grade 4/6 systolic murmur at the left lower sternal border. Vital signs on admission were blood pressure of 160/55 mmHg, pulse rate of 80 bpm, temperature of 97.3°F, and respiratory rate of 18 breaths/minute. Laboratory results indicated leukocytosis (18.59 K/uL), elevated serum potassium (5.7 mmol/L), hyponatremia (132 mmol/L), elevated lactate (8.3 mmol/L), elevated thyroid-stimulating hormone (7 mIU/L), and free T4 (4.1 ng/dL). Troponin T was elevated at 0.107 ng/mL (reference range: <0.010 ng/mL), with an electrocardiogram showing normal sinus rhythm (Figure 1). Chest X-ray revealed a globular enlargement of the cardiac silhouette suggesting pericardial effusion (Figure 2). Point-of-care ultrasound performed by the ED physicians confirmed the presence of a large pericardial effusion. Four hours after arrival at the ED, the patient experienced sudden-onset abdominal pain and nausea. He was noted to have elevated liver enzymes, with aspartate aminotransferase (AST) at 3679 U/L, alanine aminotransferase (ALT) at 2281 U/L, alkaline phosphatase at 219 U/L, and total bilirubin at 1.0 mg/dL. Eight hours after arrival, the patient became hypothermic to 94.1°F and hypoxic to oxygen saturation (SpO2) of 84%, which prompted 6 L oxygen supplementation via nasal cannula. Empiric antibiotics were initiated because of concerns for sepsis due to intra-abdominal pathology, and morphine was administered for pain relief. Abdominal and pelvic CT scans revealed moderate abdominopelvic ascites, cholelithiasis, gallbladder wall thickening, adenomatosis, and increased liver echogenicity in addition to large pericardial effusion. On hospital day two, one liter of fluid was removed via hemodialysis due to hyperkalemia (7.3 mmol/L) and increased abdominal ascites. A transthoracic echocardiogram obtained on the evening of hospital day two showed left ventricular ejection fraction of 55%, left atrium diameter of 3.6 cm, left ventricle diameter during diastole of 3.5 cm, left ventricle diameter during systole of 2.6 cm, sclerotic aortic valve leaflets, large pericardial effusion, right ventricle with free wall collapse, and mild right atrial chamber collapse consistent with tamponade physiology (Figures 3-5). Cardiothoracic surgery was consulted for surgical pericardiotomy; however, their earliest availability was in two days. Due to the concerns for cardiac tamponade, the patient was recommended to be immediately transferred to a facility with a cardiac intensive care unit (CICU). Upon arrival of transportation on hospital day three to move the patient to a CICU-capable hospital, the patient was noted to be obtunded. Point-of-care glucose at the time was 47 mg/dL and he was given a 50% dextrose injection. Vital signs at the time were as follows: blood pressure of 153/74 mmHg, pulse rate of 50 bpm, and SpO2 of 90% on 4 L oxygen. The patient was subsequently noted to desaturate to SpO2 of 75% and was without pulse. He was noted to be in asystole. Cardiopulmonary resuscitation (CPR) was initiated. Pericardiocentesis was performed by trauma surgery, extracting a total of 250 ml of bloody fluid, and a pericardial drain was placed. Despite multiple rounds of CPR, a return of spontaneous circulation was not achieved. The patient was pronounced dead from cardiac arrest due to pericardial tamponade.

## Discussion

Cardiac tamponade, a rare but life-threatening medical emergency, occurs when there is a significant accumulation of fluid within the pericardial sac, leading to an increase in intrapericardial pressure and compression of the heart chambers. The clinical presentation of cardiac tamponade can be subtle and nonspecific, making it challenging to diagnose in its early stages. Cardiac tamponade necessitates swift identification and treatment as a clinical diagnosis to avert cardiovascular failure and cardiac arrest [1,2].

This case report highlights the potential consequences of acute on chronic pericardial effusion, where an already existing pericardial effusion becomes exacerbated, culminating in a fatal outcome.

Pericardial effusion refers to the unusual buildup of fluid within the pericardial cavity. In a healthy person, the pericardial sac typically holds around 15 to 50 milliliters of serous fluid [3,4]. Chronic pericardial effusion, defined as fluid accumulation within the pericardial sac persisting for more than three months, often develops gradually, allowing the pericardium to stretch and accommodate the accumulating fluid without causing significant symptoms [4]. Patients may remain asymptomatic for an extended period until the effusion reaches a critical volume, precipitating acute decompensation. In this case, the patient's asymptomatic chronic pericardial effusion concealed the underlying disease process until the acute event, leading to cardiac tamponade.

Pericardial effusion causing tamponade has the potential to arise in patients with nearly any condition that impacts the pericardium. Among the groups of patients with a greater occurrence of pericardial effusion, individuals in the advanced stages of renal disease can be included. In some instances, a clear etiology may not be identified, leading to the classification of idiopathic pericardial effusion [5,6]. In the patient under consideration, the cause of the chronic pericardial effusion may be related to uremia or other factors, and subsequent hemodialysis may be the acute event that precipitated a rapid accumulation of pericardial fluid leading to cardiac tamponade. This patient did receive hemodialysis on hospital day two. The clinical presentation most often includes dyspnea, chest pain, and fatigue. In this case, a middle-aged male with a complex medical history presented with generalized weakness, fatigue, and right shoulder pain. Despite his intricate medical background, including cerebral palsy, hypertension, subclinical hypothyroidism, thyroid disease, and end-stage renal disease on hemodialysis, the evolution of his chronic pericardial effusion to life-threatening tamponade physiology was rapid and striking.

The patient's history of chronic pericardial effusion highlights the challenges associated with diagnosing and managing this condition. Chronic pericardial effusion can evolve insidiously, often remaining clinically silent until complications such as tamponade arise. This patient, who was being evaluated for sepsis, was hemodynamically stable for his entire hospital course up until the point he went into asystole. Furthermore, distinguishing between chronic and acute pericardial effusion can be challenging due to the subtle nature of symptom progression.

The development of tamponade physiology is a critical juncture in pericardial effusion progression. The clinical manifestations of tamponade, including reduced cardiac output, elevated venous pressures, and impaired filling of cardiac chambers, can lead to hemodynamic compromise and potentially fatal consequences. The swift transition from the stable pericardial effusion observed in previous imaging to tamponade physiology underscores the dynamic nature of this condition [7].

This case also highlights the importance of employing various diagnostic tools to ascertain the severity of pericardial effusion and its associated complications. Chest X-rays, echocardiograms, and ultrasound were pivotal in diagnosing pericardial effusion and its progression to tamponade. The diagnostic echocardiogram results observed in the patient comprise a substantial pericardial effusion, distinctive right ventricular free wall collapse indicative of tamponade, and slight collapse of the right atrial chamber, which is a responsive indicator. Another potential responsive sign, although not detailed in this case, is the existence of a distended, non-collapsible inferior vena cava (IVC) [8]. These imaging modalities, along with clinical assessment and laboratory findings, provided critical insights for informed decision-making.

The swift and severe decompensation in this case emphasizes the necessity of early intervention for patients with chronic pericardial effusion. The sudden onset of symptoms, rapid progression to tamponade, and ultimate fatal outcome serve as a somber reminder of the critical importance of closely monitoring patients with known pericardial effusions, especially in the presence of risk factors such as chronic medical conditions.

This case report has limitations. The patient's unique medical history and complexities might limit the generalizability of the findings. Additionally, factors contributing to the rapid progression from effusion to tamponade, such as the presence of concurrent medical conditions, require further investigation. Other limitations include delay in obtaining an echocardiogram until hospital day two and having to rely on point-of-care ultrasound prior to that. There was also a delay in obtaining an operating room for surgical pericardiotomy and a delay in obtaining transport to a hospital with a CICU.

## Conclusions

The index case presented emphasizes the dynamic and potentially fatal progression of pericardial effusion to tamponade physiology, especially in patients with chronic medical conditions and comorbidities. As evidenced in this instance, the rapid evolution to tamponade can be imminent in such patients, underscoring the need for risk stratification. Healthcare providers must exercise heightened vigilance when assessing patients with chronic pericardial effusion and consider early intervention upon noting signs of progression. Embracing an individualized and etiologically driven approach, leveraging advancements in diagnostic tools, fostering interdisciplinary collaboration, and ensuring swift clinical decision-making are pivotal in averting disastrous outcomes. Further research is imperative to delve deeper into the predictors and mechanisms that accelerate the transition of pericardial effusion to tamponade. This will facilitate the formulation of more potent strategies for timely intervention and prevention.
